# Supplementary material for: 5-hydroxymethylcytosine marks regions with reduced mutation frequency in human DNA
Source: eLife. 2016 May 16;5:e17082. doi: 10.7554/eLife.17082 (PMC4931910; doi:10.7554/eLife.17082)
Supplement: Supplementary file 1. — (a) Overview of BS-Seq and TAB-Seq data used to generate modification maps. (b) Overview of whole genome and exome sequencing data used for mutation information. DOI: http://dx.doi.org/10.7554/eLife.17082.021 [file elife-17082-supp1.docx]

Supplementary file 1a: Overview of BS-Seq and TAB-Seq data used to generate modification maps.

| **Tissue** | **BS-Seq** |  | **TAB-Seq** |  | **Source** | **Link** |
| --- | --- | --- | --- | --- | --- | --- |
|  | CpGs | Average | CpGs | Average |  |  |
| **Brain** | 53388534 | 0.791200 | 53847986 | 0.221845 | (Wen et al., 2014) | [BS](http://www.ncbi.nlm.nih.gov/geo/query/acc.cgi?acc=GSM1135081), [TAB](http://www.ncbi.nlm.nih.gov/geo/query/acc.cgi?acc=GSM1135082) |
| **Kidney r1** | 54117976 | 0.759816 | 46303252 | 0.088314 | (Chen et al., 2015) | [BS](http://www.ncbi.nlm.nih.gov/geo/query/acc.cgi?acc=GSM1546664), [TAB](http://www.ncbi.nlm.nih.gov/geo/query/acc.cgi?acc=GSM1546660) |
| **Kidney r2** | 53861360 | 0.756454 | 54585341 | 0.094624 | (Chen et al., 2015) | [BS](http://www.ncbi.nlm.nih.gov/geo/query/acc.cgi?acc=GSM1546666), [TAB](http://www.ncbi.nlm.nih.gov/geo/query/acc.cgi?acc=GSM1546662) |
| **Kidney total** | 54857866 | 0.757601 | 54928295 | 0.092868 | (Chen et al., 2015) |  |
| **Blood dendritic r1** | 24586388 | 0.791371 | - | - | (Pacis et al., 2015) | [BS](http://www.ncbi.nlm.nih.gov/geo/query/acc.cgi?acc=GSM1565940) |
| **Blood dendritic r2** | 23524704 | 0.786322 | - | - | (Pacis et al., 2015) | [BS](http://www.ncbi.nlm.nih.gov/geo/query/acc.cgi?acc=GSM1565942) |
| **Blood dendritic r3** | 24419613 | 0.782467 | - | - | (Pacis et al., 2015) | [BS](http://www.ncbi.nlm.nih.gov/geo/query/acc.cgi?acc=GSM1565944) |
| **Blood dendritic r4** | 24452547 | 0.787728 | - | - | (Pacis et al., 2015) | [BS](http://www.ncbi.nlm.nih.gov/geo/query/acc.cgi?acc=GSM1565946) |
| **Blood dendritic r5** | 24399654 | 0.774058 | - | - | (Pacis et al., 2015) | [BS](http://www.ncbi.nlm.nih.gov/geo/query/acc.cgi?acc=GSM1565948) |
| **Blood dendritic r6** | 24533745 | 0.790145 | - | - | (Pacis et al., 2015) | [BS](http://www.ncbi.nlm.nih.gov/geo/query/acc.cgi?acc=GSM1565950) |
| **Blood dendritic total** | 25586845 | 0.789083 | 27754454 | 0.029103 | (Pacis et al., 2015) | [TAB](http://www.ncbi.nlm.nih.gov/geo/query/acc.cgi?acc=GSM1565996) |
| **Breast** | 53222114 | 0.735273 | - | - | Epigenome Roadmap | [BS](ftp://ftp.ncbi.nlm.nih.gov/geo/samples/GSM1127nnn/GSM1127125/suppl/GSM1127125_UCSF-UBC.Breast_Luminal_Epithelial_Cells.Bisulfite-Seq.RM066.wig.gz) |
| **Pancreas** | 54341922 | 0.697484 | - | - | Epigenome Roadmap | [BS](ftp://ftp.ncbi.nlm.nih.gov/geo/samples/GSM983nnn/GSM983651/suppl/GSM983651_UCSD.Pancreas.Bisulfite-Seq.STL003.wig.gz) |
| **Lung** | 54236520 | 0.774050 | - | - | Epigenome Roadmap | [BS](ftp://ftp.ncbi.nlm.nih.gov/geo/samples/GSM983nnn/GSM983647/suppl/GSM983647_UCSD.Lung.Bisulfite-Seq.STL002.wig.gz) |
| **Liver** | 51884076 | 0.757123 | - | - | Epigenome Roadmap | [BS](ftp://ftp.ncbi.nlm.nih.gov/geo/samples/GSM916nnn/GSM916049/suppl/GSM916049_BI.Adult_Liver.Bisulfite-Seq.3.wig.gz) |
| **Stomach** | 54054176 | 0.762082 | - | - | Epigenome Roadmap | [BS](ftp://ftp.ncbi.nlm.nih.gov/geo/samples/GSM1010nnn/GSM1010984/suppl/GSM1010984_UCSD.Gastric.Bisulfite-Seq.STL003.wig.gz) |
| **Blood (hematopoietic multipotent progenitor cell)** | 51822931 | 0.852170 | - | - | Blueprint Epigenome | [BS](http://ftp.ebi.ac.uk/pub/databases/blueprint/data/homo_sapiens/GRCh38/Bone_marrow/F2012-2912/hematopoietic_multipotent_progenitor_cell/Bisulfite-Seq/CNAG/HPC-V151.CPG_methylation_calls.bs_call.GRCh38.20150707.bw) |

Supplementary file 1b: Overview of whole genome and exome sequencing data used for mutation information.

| **Cancer type** | **Exome** | | **Whole genome** | | **Tissue** | **Source** |
| --- | --- | --- | --- | --- | --- | --- |
|  | Patients | SNVs | Patients | SNVs |  |  |
| **Glioblastoma** | 39 | 22954 | - | - | brain | (Alexandrov et al., 2013) |
| **Glioma Low Grade** | 215 | 9678 | - | - | brain | (Alexandrov et al., 2013) |
| **Medulloblastoma** | - | - | 100 | 139553 | brain | (Alexandrov et al., 2013) |
| **Neuroblastoma** | 210 | 5027 | - | - | brain | (Alexandrov et al., 2013) |
| **Pilocytic Astrocytoma** | - | - | 101 | 12989 | brain | (Alexandrov et al., 2013) |
| **Kidney Chromophobe** | 65 | 1646 | - | - | kidney | (Alexandrov et al., 2013) |
| **Kidney Clear Cell** | 325 | 30273 | - | - | kidney | (Alexandrov et al., 2013) |
| **Kidney Papillary** | 100 | 6479 | - | - | kidney | (Alexandrov et al., 2013) |
| **RECA-EU** | - | - | 95 | 488922 | kidney | ICGC |
| **Acute Myeloid Leukaemia** | 147 | 2214 | 7 | 3659 | blood myeloid | (Alexandrov et al., 2013) |
| **Acute Myeloid Leukaemia** | - | - | 49 | 176164 | blood myeloid | TCGA |
| **Acute Lymphoblastic Leukaemia** | 140 | 1869 | 1 | 7881 | blood other | (Alexandrov et al., 2013) |
| **Chronic Lymphoid Leukaemia** | 103 | 3998 | 28 | 54746 | blood other | (Alexandrov et al., 2013) |
| **Lymphoma B-cell** | 24 | 824 | 24 | 142753 | blood other | (Alexandrov et al., 2013) |
| **Myeloma** | 69 | 3973 | - | - | blood other | (Alexandrov et al., 2013) |
| **Breast** | 844 | 55731 | 119 | 647692 | breast | (Alexandrov et al., 2013) |
| **Liver** | - | - | 88 | 899445 | liver | (Alexandrov et al., 2013) |
| **Lung Adeno** | 636 | 248519 | 24 | 1505512 | lung | (Alexandrov et al., 2013) |
| **Lung Small Cell** | 70 | 17639 | - | - | lung | (Alexandrov et al., 2013) |
| **Lung Squamous** | 176 | 70485 | - | - | lung | (Alexandrov et al., 2013) |
| **Pancreas** | 98 | 5093 | 15 | 122787 | pancreas | (Alexandrov et al., 2013) |
| **Stomach** | 212 | 102110 | 0 | 0 | stomach | (Alexandrov et al., 2013) |
| **Stomach** | 0 | 0 | 100 | 1995618 | stomach | (Wang et al., 2014) |
